# Homozygous loss-of-function variants in *FILIP1* cause autosomal recessive *arthrogryposis multiplex congenita* with microcephaly

**DOI:** 10.1007/s00439-023-02528-2

**Published:** 2023-03-21

**Authors:** Franziska Schnabel, Elisabeth Schuler, Almundher Al-Maawali, Ankur Chaurasia, Steffen Syrbe, Adila Al-Kindi, Gandham SriLakshmi Bhavani, Anju Shukla, Janine Altmüller, Peter Nürnberg, Siddharth Banka, Katta M. Girisha, Yun Li, Bernd Wollnik, Gökhan Yigit

**Affiliations:** 1 Institute of Human Genetics, University Medical Center Göttingen, Heinrich-Düker-Weg 12, 37073 Göttingen, Germany; 2 Institute of Human Genetics, University of Leipzig Hospitals and Clinics, 04103 Leipzig, Germany; 3 Division of Paediatric Epileptology, Centre for Paediatrics and Adolescent Medicine, University Hospital Heidelberg, Im Neuenheimer Feld 430, 69120 Heidelberg, Germany; 4 Department of Genetics, College of Medicine and Health Sciences, Sultan Qaboos University, Muscat, Oman; 5 Genetic and Developmental Medicine Clinic, Sultan Qaboos University Hospital, Muscat, Oman; 6 Department of Medical Genetics, Kasturba Medical College, Manipal, Manipal Academy of Higher Education, Manipal, India; 7 Division of Evolution, Infection and Genomics, School of Biological Sciences, Faculty of Biology, Medicine and Health, University of Manchester, Manchester, M13 9PL UK; 8 Cologne Center for Genomics (CCG), Faculty of Medicine and University Hospital Cologne, University of Cologne, Cologne, Germany; 9 Core Facility Genomics, Berlin Institute of Health at Charité - Universitätsmedizin Berlin, Berlin, Germany; 10 Max Delbrück Center for Molecular Medicine in the Helmholtz Association (MDC), Berlin, Germany; 11 Center for Molecular Medicine Cologne (CMMC), Faculty of Medicine and University Hospital Cologne, University of Cologne, Cologne, Germany; 12 Manchester Centre for Genomic Medicine, Health Innovation Manchester, St Mary’s Hospital, Manchester University NHS Foundation Trust, Manchester, M13 9WL UK; 13 Cluster of Excellence “Multiscale Bioimaging: From Molecular Machines To Networks of Excitable Cells” (MBExC), University of Göttingen, 37073 Göttingen, Germany; 14 DZHK (German Centre for Cardiovascular Research), Partner Site Göttingen, Göttingen, Germany

## Abstract

*Arthrogryposis multiplex congenita* forms a broad group of clinically and etiologically heterogeneous disorders characterized by congenital joint contractures that involve at least two different parts of the body. Neurological and muscular disorders are commonly underlying arthrogryposis. Here, we report five affected individuals from three independent families sharing an overlapping phenotype with congenital contractures affecting shoulder, elbow, hand, hip, knee and foot as well as scoliosis, reduced palmar and plantar skin folds, microcephaly and facial dysmorphism. Using exome sequencing, we identified homozygous truncating variants in *FILIP1* in all patients. FILIP1 is a regulator of filamin homeostasis required for the initiation of cortical cell migration in the developing neocortex and essential for the differentiation process of cross-striated muscle cells during myogenesis. In summary, our data indicate that bi-allelic truncating variants in *FILIP1* are causative of a novel autosomal recessive disorder and expand the spectrum of genetic factors causative of *arthrogryposis multiplex congenita*.

## Introduction

*Arthrogryposis multiplex congenita* (AMC) refers to a wide range of congenital conditions defined by joint contractures in at least two body areas (Cachecho et al. 2019). The overall prevalence is estimated at about 1/3000–1/5000 (Lowry et al. 2010; Le Tanno et al. 2021) and the underlying etiology mainly includes impairment in muscle, central and peripheral nervous system, neuromuscular junction, connective tissue and metabolic pathways as well as extrinsic factors such as uterine space limitations, maternal diseases and drug intake (Hall 2014; Hall et al. 2019). Reduced fetal movement is a common feature in all conditions leading to connective tissue depositions around the joints, disuse muscle atrophy and abnormal joint surface all resulting in restricted joint movements (Hall 2014). Additional clinical aspects include polyhydramnios, intrauterine growth restriction, craniofacial dysmorphism, pulmonary hypoplasia and skin anomalies (Hall 2014; Le Tanno et al. 2021). Although more than 400 genes have been associated with arthrogryposis until now, the molecular pathogenesis still remains unclear in a large number of cases (Kiefer and Hall 2019). Generally, it is considered that genetic or environmental factors that restrict or interfere with fetal movement may lead to arthrogryposis. Genetic factors causing arthrogryposis include genes involved in central and peripheral nervous system development as well as genes associated with muscular disorders and impaired development of connective tissues (Hall et al. 2019). Genetic counseling and diagnostics of AMC are further complicated by the fact that AMC shows a broad range of intra- and interfamilial variability regarding both, the position contractures take place as well as severity of symptoms.

Filamin-A-interacting protein 1 (FILIP1) has first been identified as a protein regulating the start of cortical cell migration in the developing neocortex through filamin A (FLNa) degradation. In this function, FILIP1 induces degradation of FLNa and suppresses radial cell migration out of the ventricular zone in neocortical neurons (Nagano et al. 2002). FILIP1 and FLNa also have an influence on cell polarity and motility in migrating neocortical neurons (Nagano et al. 2004). Interestingly, expression of *FILIP1* has not only been observed in the central nervous system, but a robust gene expression has also been detected in heart, skeletal and smooth muscle, suggesting that FILIP1 might also be involved in myogenesis (Nagano et al. 2002; Sato and Nagano 2005).

Here, we report five affected children from three independent families originating from Pakistan, Oman, and India sharing multiple joints contractures, scoliosis, reduced palmar and plantar skin folds, microcephaly and facial dysmorphism. In an exome sequencing (ES) approach, we were able to show that all affected individuals carry homozygous truncating variants in *FILIP1,* providing evidence that bi-allelic loss of its protein function leads to AMC in humans.

## Materials and methods

### Patients

**Fig. 1:**
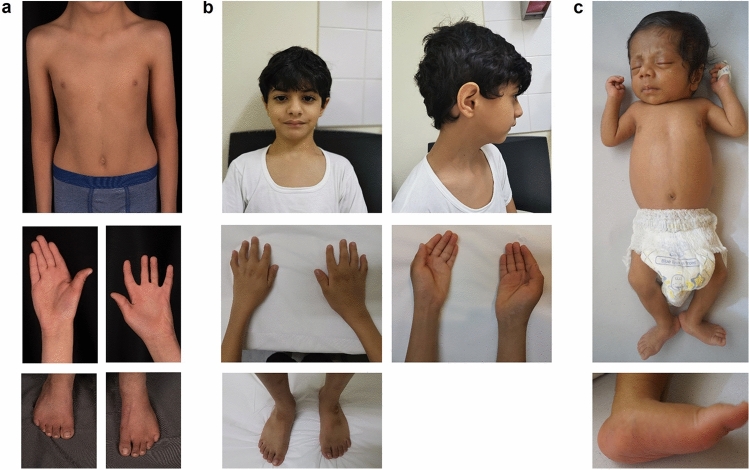
Clinical presentation of patients with homozygous *FILIP1* variants. **a** Individual III.2 of family 1 at 6 years presenting with webbed neck, camptodactyly, an operated left club foot and an equinus position of the right foot. **b** Individual III.3 of family 2 at 7 years presenting with triangular face, low anterior hairline, downslanted palpebral fissures and micrognathia as well as camptodactyly, cutaneous syndactyly of second and third toes, ulnar deviation and club feet. **c** Individual III.3 of family 3 at 28 days with tall forehead, long philtrum, anteverted nares, short neck, overlapping fingers and rocker bottom foot

All five affected individuals reported herein were born to healthy, consanguineous parents and presented with an overlapping arthrogryposis phenotype. We used the GeneMatcher (Sobreira et al. 2015) tool to connect the three centers at the University Medical Center Göttingen (Göttingen, Germany), the Sultan Qaboos University Hospital (Muscat, Oman) and the Kasturba Medical College (Manipal, India), in which clinical examination of patients and/or genetic analyses were performed. Written informed consent for genetic diagnostics and for publication of clinical data and genetic results was obtained from all subjects or their legal representatives. The authors affirm that human research participants provided informed consent for publication of the images in Fig. 1a–c. The study was conducted in accordance with the Declaration of Helsinki protocols and approved by the local institutional ethics board (No 3/2/16, University Medical Center Göttingen, Germany).

### Genetics analysis

In family 1, we performed ES on genomic DNA extracted from peripheral blood lymphocytes of the affected brothers III.1 and III.2. Exonic and adjacent intronic sequences were enriched using the Agilent SureSelect Human All Exon V6 enrichment kit and were run on an Illumina HiSeq4000 sequencer. Data analysis and filtering of mapped target sequences were conducted by the exome and genome analysis pipeline “Varbank 2.0” (https://varbank.ccg.uni-koeln.de/varbank2/) of the Cologne Center for Genomics (CCG, University of Cologne, Germany). We obtained a mean coverage of 58–63 reads, and 95.8–96.2% of targets were covered more than 10x. Sanger sequencing was used to validate the homozygous variant in the three affected brothers and the heterozygous carrier state in both parents.

In family 2, ES of patient III.3 was performed on genomic DNA using the Agilent SureSelectXT Human All Exon 50 Mb enrichment kit on an Illumina HiSeq4000 sequencer. ES data analysis and filtering of variants were carried out by an in-house filter pipeline at the Genome Diagnostics Nijmegen (Radboud University Medical Center, Netherlands). Variants were assessed for clinical phenotypic match and according to the American College of Medical Genetics and Genomics (ACMG) guidelines (Richards et al. 2015). Confirmation of the identified homozygous variant in the affected individual and segregation analysis in both parents and all siblings was performed by Sanger sequencing.

In family 3, we initiated trio-ES on genomic DNA obtained from peripheral blood lymphocytes of the patient III.3 and his parents. The exonic and flanking genomic regions were captured using Agilent SureSelect Clinical Research Exome V2 capture kit. Exome sequencing had an average coverage of 100 to 130 × , and 95% of bases covered at a minimum of 20 × with 90% sensitivity (Girisha et al. 2019). Raw data were retrieved in FASTQ format and aligned to GRCh38 assembly using Burrows-Wheeler Aligner (v0.7.15) and the in-house pipeline based on Genome Analysis Toolkit Best Practices. Copy number variation (CNV) calling was performed on exome data using cn.MOPS CNV calling tool (Klambauer et al. 2012). An in-house CNV frequency dataset was used to annotate the called CNVs. These CNVs were annotated for gene names, OMIM phenotypes and HPO terms. The deletions were manually inspected in IGV against 5 references (randomly selected exomes from unaffected individuals sequenced from the same capture kit). Poorly mapped reads were excluded from IGV visualization (reads with mapping quality—MAPQ—lower than 30). The CNVs were classified as either true or false calls. Quantitative PCR (qPCR) by comparative quantification Ct (∆∆Ct) method (Livak and Schmittgen 2001) was used for validation of results obtained from exome sequencing data performed on genomic DNA of the patient and his parents for the putative target region (exons 4, 5 of *FILIP1* and exon 2 of *FILIP1* for control) using Applied Biosystems StepOne™ Real-Time PCR System, PowerUp SYBR Green PCR Master Mix and StepOne Software v2.3 for data analysis. The relative exon copy number was calculated by the expression 2 × 2^(−ΔΔCt)^.

## Results

### Clinical description

**Fig. 2:**
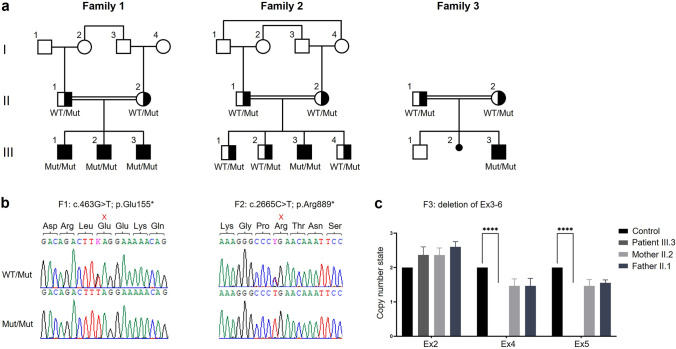
Pedigrees and genetic characteristics of individuals with congenital arthrogryposis and microcephaly carrying homozygous disease-causing variants in *FILIP1*. **a** Pedigrees of families 1–3 with pathogenic *FILIP1* variants. Affected siblings (solid symbols) in each family carry homozygous disease-causing variants in *FILIP1* while non-affected parents and siblings (semi-solid symbols) are heterozygous for identified *FILIP1* variants. **b** Chromatograms of the identified *FILIP1* variants in family 1 (F1: c.463G>T; p.Glu155*) and family 2 (F2: c.2665C>T; p.Arg889*) showing homozygosity in affected patients (Mut/Mut) and heterozygous carrier state in healthy parents or siblings (WT/Mut). Localization of nonsense variants is indicated in red. **c** Copy number analysis by qPCR was used for segregation analysis of a ~ 86-kb homozygous deletion spanning *FILIP1* exons 3–6 in family 3 (F3: deletion of Ex3–6) which was initially detected in exome sequencing data of the patient. qPCR for exons 4 and 5 confirmed homozygous deletion of this region in the patient and a heterozygous carrier state in his parents compared to exon 2, which was used as reference

Family 1 is a consanguineous family with the 12-, 6- and 5-year-old brothers III.1, III.2 and III.3 referred for medical evaluation due to multiple congenital joint contractures (Fig. 2a, Table 1). Pregnancy history and birth parameters were not available. Individual III.1 showed movement restriction of shoulder, knee and ankle joints as well as limitations in elbow flexion, internal hip and head rotation. Additional clinical features included microcephaly, camptodactyly, scapular winging, scoliosis, pectus excavatum, reduced palmar and plantar skin folds and translucent skin. Due to finger contractures, surgery was performed. Motor development was generally normal, but reduced fine motor skills and poor active speech were observed. Spinal MRI revealed a cleft formation in the anterior atlas arch. Facial dysmorphism included long face, downslanted palpebral fissures, bifid uvula, high narrow palate, micrognathia and webbed neck. At the last follow-up at the age of 12 years, his weight was 35.5 kg (− 1.0 SD), his length was 145 cm (− 1.1 SD) and his head circumference was 51.8 cm (− 2.1 SD). Physical examination of individual III.2 revealed limitations in shoulder, elbow extension, hip, knee and head rotation, camptodactyly and shortening of the entire dorsal leg muscles with a left club foot and an equinus position of the right foot. Club foot was operated on at the age of 5 years. Similarly to his affected brother III.1, he presented with long face, downslanted palpebral fissures, bifid uvula, high narrow palate, micrognathia, webbed neck, scapular winging, scoliosis, reduced palmar and plantar skin folds and translucent skin (Fig. 1a). Motor and speech development were mildly delayed. Growth measurements at the age of 6 years revealed a body weight of 22.0 kg (− 0.1 SD), a body length of 127 cm (1.4 SD) and a head circumference of 51.0 cm (− 1.5 SD). Individual III.3 showed a less severe phenotype than his brothers. He also presented with movement restrictions mainly in shoulder and elbow, whereas hip and knee are mildly affected. Additional clinical characteristics included scoliosis, camptodactyly, reduced palmar and plantar skin folds, translucent skin as well as congenital renal hypoplasia of the left side. Motor and speech development were mildly delayed. Craniofacial features were similar to those of his brothers with long face, downslanted palpebral fissures, mild micrognathia and webbed neck. Cardiac investigations in all affected brothers revealed no anomalies.

**Table 1:** Clinical features of patients with disease-causing variants in *FILIP1*

Family	Family 1	Family 1	Family 1	Family 2	Family 3
Pedigree ID	III.1	III.2	III.3	III.3	III.3
Gender	Male	Male	Male	Male	Male
Geographic origin	Pakistan	Pakistan	Pakistan	Oman	India
Consanguinity	+	+	+	+	+
*FILIP1* variant (all in homozygous state)	c.463G>T (p.Glu155*)	c.463G>T (p.Glu155*)	c.463G>T (p.Glu155*)	c.2665C>T (p.Arg889*)	Deletion of exons 3–6
Age at examination	12 years	6 years	5 years	7 years	11 months
Length	145 cm (− 1.1 SD)	127 cm (1.4 SD)	N/A	123 cm (− 0.6 SD)	66 cm (− 3.2 SD)
Weight	35.5 kg (− 1.0 SD)	22.0 kg (− 0.1 SD)	N/A	19.9 kg (− 1.7 SD)	6.7 kg (− 3.5 SD)
Head circumference	51.8 cm (− 2.1 SD)	51.0 cm (− 1.5 SD)	N/A	48.5 cm (− 3.2 SD)	42.5 cm (− 4.0 SD)
Clinical characteristics					
Shoulder contractures	+	+	+	−	−
Elbow contractures	+	+	+	+	+
Wrist contractures	+	+	+	+	−
Camptodactyly	+	+	+	+	+
Overlapping fingers	−	−	−	−	+
Syndactyly	−	−	−	+	−
Hip contractures	+	+	+	−	−
Knee contractures	+	+	+	−	+
Foot deformity (club or rocker bottom foot)	−	+	−	+	+
Scoliosis	+	+	+	+	−
Reduced palmar and plantar skin folds	+	+	+	+	N/A
Translucent skin with prominent veins	+	+	+	−	+
Facial dysmorphism	+	+	+	+	+
Heart anomalies	−	−	−	−	−

*N/A* not available, *SD* standard deviations

Family 2 is a consanguineous family from Oman. Individual III.3 is a 7-year-old male and was born as the third child to double cousin parents (Fig. 2a, Table 1). During pregnancy, the mother noted decreased fetal movements, otherwise pregnancy and delivery were unremarkable. His birth weight was 2.9 kg (− 1.3 SD), his birth length 56 cm (1.8 SD), and his head circumference 33 cm (− 1.8 SD). Arthrogryposis affecting elbow, wrist, fingers and feet was noted on both sides. In addition, he presented with muscular hypotonia, kyphoscoliosis, reduced palmar and plantar skin folds as well as club feet. Facial dysmorphism included facial hypotonia, triangular facies, low anterior hairline, downslanted palpebral fissures and micrognathia (Fig. 1b). During early childhood, he did not have any major developmental delay apart from difficulties in using his hands. He started sitting without support at the age of 10 months, walking without support at the age of 16 months and spoke his first words at the age of 12 months. Stanford Binet IQ assessment (Janzen et al. 2004) at the age of 5 years showed normal mental function. Electrocardiogram revealed a normal sinus rhythm with normal interval and repolarization, while echocardiography showed normal ejection fraction and mild left ventricular dilation. Investigations of the patient including routine blood investigations, CK levels, liver function tests, brain imaging, and chromosomal microarray were within normal limits. Currently, his weight is 19.9 kg (− 1.7 SD), his length is 123 cm (− 0.6 SD) and his head circumference is 48.5 cm (− 3.2 SD).

Family 3 is of Indian origin and the parents are consanguineous. Individual III.3 is the third child of healthy parents (Fig. 2a, Table 1). The mother’s previous pregnancy ended in intrauterine fetal death and oligohydramnios. After an uneventful pregnancy, the patient was born at term via lower segment cesarean section. He had a birth weight of 2.3 kg (− 2.3 SD). On day 3 of life, he was noticed to have icterus with pale stools, dark yellow colored urine, and distended gall bladder. On clinical examination at day 28 of life, his body weight was 3.5 kg (− 1.2 SD), his length was 48 cm (− 3.4 SD), his head circumference was 36 cm (− 1.2 SD). He was evaluated again at 11 months of life when he had a weight of 6.7 kg (− 3.5 SD), a length of 66 cm (− 3.2 SD) and head circumference of 42.5 cm (− 4.0 SD). He attained neck holding by 3 months of age, sitting without support by 9 months of age, and walking without support by age 1 year 6 months with ability to climb five to six steps. He was able to speak monosyllables by 9 months and bisyllables by 10 months. At 1 year and 10 months of age, it was noted that he can speak a few words but cannot form full sentences. Moreover, he presented with microcephaly, limited movement of wrist and elbow joints, and rocker bottom feet due to which he has an impaired gait (Fig. 1c). Facial features involve epicanthal folds, prominent nose with a broad tip, anteverted nares, long and broad philtrum, short columella, small mouth, and short and broad neck. In addition, prominent veins over abdomen and cholestatic jaundice with scleral icterus were noted. Biochemical blood investigations revealed increased levels of total and direct bilirubin, serum aspartate transaminase, serum alanine transaminase and alkaline phosphatase as well as macrocytic anemia and neutrophilic leukocytosis. Cardiac investigation including electrocardiogram and echocardiography was normal.

### Genetic results

**Fig. 3:**
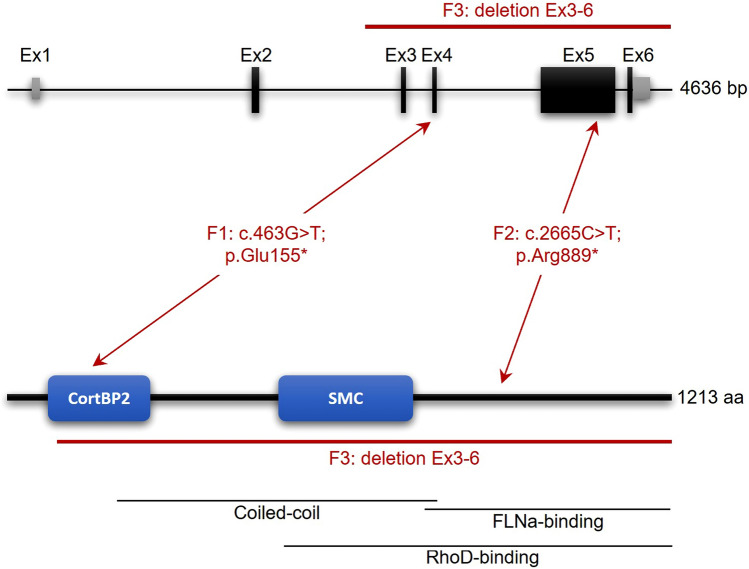
Schematic representation of the genomic (upper panel) and protein structure (lower panel) of FILIP1. Introns are shown by black horizontal line, coding exons by black bars, non-coding regions of exons by grey bars (upper panel). FILIP1 protein contains a cortactin-binding protein-2 domain (CortBP2) and a central structural maintenance of chromosome (SMC) domain as well as FLNa- and RhoD-binding motifs (lower panel). Localization of the identified nonsense variants in families 1 and 2 (F1; F2) and the deletion of exons 3–6 in family 3 (F3) is marked in red on genomic (RefSeq NM_015687.4) and protein level (RefSeq NP_056502.1)

In family 1, based on the parental consanguinity, we focused on homozygous variants and filtered the ES data of the two affected brothers III.1 and III.2 for variants with coverage of more than 6 reads, a minimum quality score of 10, an allele frequency ≥ 75%, a minor allele frequency (MAF) < 1.0% in the gnomAD database (http://gnomad.broadinstitute.org/), and not annotated in the in-house WES datasets of the CCG. Using these filter criteria, we identified only one homozygous truncating variant shared by the bothers III.1 and III.2 that is predicted to have a severe impact on protein function and to be most likely deleterious. The variant, c.463G>T, is located in exon 4 of the *FILIP1* gene (GenBank: NM_015687.4) and induces the formation of a premature stop codon at the amino acid position 155 (p.(Glu155*)) (Fig. 3). The variant is absent from gnomAD database and is classified as pathogenic according to ACMG guidelines (Richards et al. 2015; criteria PVS1, PM2_sup, PM3_sup). In line with the reported parental consanguinity, *FILIP1* is embedded within a homozygous stretch of 56.5 Mb (located between positions chr6:36,765,355 and chr6:93,257,601) in individual III.1 and a homozygous stretch of 29.2 Mb (located between positions chr6:49,969,544 and chr6:79,202,472) in individual III.2. We confirmed the presence of the homozygous variant in all three affected brothers III.1, III.2 and III.3 as well as the heterozygous carrier status of the parents by Sanger sequencing (Fig. 2b).

In family 2, after exclusion of pathogenic or likely pathogenic variants in any of the known genes associated with Mendelian-inherited disorders, ES data of patient III.3 was subsequently analyzed for novel putative causative variants. This analysis revealed the homozygous variant c.2665C>T in *FILIP1* that induces the formation of a premature stop codon at the amino acid position 889 (p.(Arg889*)) in exon 5 (Fig. 3). The variant was described in 3/251412 alleles (MAF = 0.00001193) in the gnomAD database, but only present in heterozygous state. Based on ACMG guidelines (Richards et al. 2015; criteria PVS1, PM2_sup, PM3_sup), the variant was classified as pathogenic. We confirmed the homozygous variant in individual III.3 by Sanger sequencing, and co-segregation analysis revealed heterozygous carrier status of both parents and all healthy siblings (Fig. 2b).

In family 3, clinically significant single nucleotide variations or small insertion-deletions correlating with the observed phenotype were not detected in exome sequencing analysis of patient III.3. Interestingly, CNV analysis revealed a ~ 86-kb homozygous deletion (chr6:75,288,632–75,374,566) spanning *FILIP1* exons 3–6 (Fig. 3). The deletion is not annotated in the Decipher database (https://decipher.sanger.ac.uk/). In accordance with the ACMG guidelines (Richards et al. 2015; criteria PVS1, PM2_sup, PM3_sup), the deletion was classified as pathogenic. Quantitative PCR showed no amplification of *FILIP1* exons 4 and 5 in the patient confirming the homozygous pattern of the deletion, while amplification values in the parents corresponded to one copy number in a heterozygous deletion. No significant differences in amplification values were detected in *FILIP1* exon 2 between the patient, his parents and the unrelated control (Fig. 2c).

## Discussion

In this study, we describe a novel arthrogryposis phenotype in five patients from three unrelated families characterized by variable severity of multiple congenital joint contractures and associated with homozygous putatively deleterious variants in the *FILIP1* gene. The phenotypic analysis of our cohort highlights that the most common clinical findings are elbow contracture, camptodactyly and feet anomalies such as club feet and rocker bottom feet present in all three families. Movement restrictions also involve shoulder, hip, knee and other large joints. Further frequent features include scoliosis, reduced palmar and plantar skin folds, translucent skin with prominent veins as well as craniofacial dysmorphism. Interestingly, three patients share microcephaly and a fourth affected individual presented with a head circumference within low standard range. Except for reduced fine motor skills due to finger contractures and gait impairment caused by feet deformities, motor development was normal. Delayed speech development was observed in the three affected individuals of family 1, whereas all other patients did not show any speech abnormalities. Overall, we observed a clinical variability even within the same family, which might be caused by modifying factors. To characterize the spectrum of phenotypic features in more detail, a larger patient cohort is needed.

Filamin-A-interacting protein 1 (FILIP1) has first been identified as a protein regulating the start of cortical cell migration in the developing neocortex through filamin A (FLNa) degradation. FILIP1 induces degradation of FLNa and suppresses radial cell migration out of the ventricular zone in neocortical neurons (Nagano et al. 2002). FILIP1 and FLNa also have an influence on cell polarity and motility in migrating neocortical neurons (Nagano et al. 2004). Interestingly, expression of *FILIP1* has not only been observed in the central nervous system (especially in amygdala and caudate nucleus), but a robust gene expression has also been detected in heart, skeletal and smooth muscle, suggesting that FILIP1 might also be involved in myogenesis. In addition, low expression levels of *FILIP1* were detected in lung, skeletal muscle, ovary, testis, kidney, and fetal brain (Nagase 1999). Expression of *FILIP1* and a functional role during tendon development are currently unclear.

FILIP1 contains a cortactin-binding protein-2 domain (CortBP2) at its N-terminus and an additional central structural maintenance of chromosome (SMC) domain (Fig. 3) (Nagano et al. 2002; Gad et al. 2012). Apart from FLNa binding, coimmunoprecipitation experiments identified the Rho family GTPase RhoD, a regulator of actin cytoskeleton dynamics mediating cell migration, cell division and vesicle trafficking, as an additional interaction partner of FILIP1 (Gad et al. 2012; Blom et al. 2017).

In this study, we identified two *FILIP1* nonsense variants and a deletion affecting exons 3–6 of *FILIP1* in patients with multiple joint contractures. The variant c.463G>T (p.(Glu155*)) in family 1 is located within the CortBP2 domain, whereas the variant c.2665C>T (p.(Arg889*)) in family 2 is related to FLNa- and RhoD-binding motifs (Fig. 3). The 86-kb deletion in family 3 affects the C-terminal part of the CortBP2 domain as well as the SMC domain and the FLNa- and RhoD-binding motifs. Homozygous truncating variants in *FILIP1* are not observed in healthy control individuals. For the canonical transcript of *FILIP1* (ENST00000237172.7, NM_015687.3) 24 alleles with nonsense variants, all in heterozygous state, were reported in the gnomAD database in contrast to 48.3 that were expected to be observed in the > 250,000 alleles. In addition, no bi-allelic CNVs encompassing *FILIP1* have been reported so far in the DECIPHER database, the Database of Genomic Variants (DGV) and the structural variant (SV) dataset of gnomAD. Until now, the functional and clinical significance of CortBP2 and SMC domains has remained unclear. Since both nonsense variants and the deletion are located within different parts of the protein and cause a similar arthrogryposis phenotype in our cohort, it is most likely that all variants induce loss of FILIP1 function, either by leading to nonsense-mediated mRNA decay, inducing protein instability and subsequent degradation of truncated FILIP1, or by functional impairment based on the loss of essential domains of FILIP1.

FILIP1 is known as a modulator of cortical cell migration during corticogenesis through the FLNa–F-actin axis. FILIP1 binding targets FLNa degradation and inhibits the start of radial cell migration from the ventricular zone (Nagano et al. 2002). FLNa is a widely expressed intracellular actin-binding protein encoded by the *FLNA* gene on the X chromosome. Remodeling actin cytoskeleton, FLNa is involved in a variety of cellular functions, such as signal transduction, cellular proliferation, differentiation and migration (Zhou et al. 2021). Alterations in the *FLNA* gene are associated with a wide phenotypic spectrum including brain periventricular nodular heterotopia (Parrini et al. 2006) and skeletal dysplasia (Moutton et al. 2016). Previous functional characterization already provided evidence that FILIP1 is involved in the regulatory network of the myogenic differentiation process where it is substantial for the formation of functional sarcomeres (Reimann et al. 2020). Myogenesis is a complex process essential for muscular tissue formation during embryonic development and for muscle regeneration in adults. Functional characterization in C2C12 myoblast cell lines supports the role of FILIP1 in regulatory networks of the myogenic differentiation process (Militello et al. 2018). Expression levels of *FILIP1* are increased during myogenic differentiation, whereas silencing of *FILIP1* impairs differentiation of myoblasts into multi-nucleated myotubes and reduces mRNA levels of myogenic regulatory factors (MRFs) *MyoD*, *Myogenin* and *Myolinc* in differentiating myoblasts (Militello et al. 2018). *Myolinc* is a long non-coding RNA which is located closely to *FILIP1* and in turn controls its expression in a cis-manner (Militello et al. 2018). *Myolinc* and *FILIP1* are both regulated by TAR DNA-binding protein 43 (TDP-43), a DNA/RNA-binding protein that is localized to the nuclei of myotubes and acts as a transcriptional regulator influencing the expression of several muscle-specific genes (Militello et al. 2018). In addition, in vivo studies in adult mice showed an increased expression of *Myolinc*, *FILIP1* and *TDP-43* during muscle regeneration (Militello et al. 2018).

Phosphoproteomics and interaction studies focusing on PI3K/Akt signaling in contracting skeletal muscle cells identified FILIP1 as a direct binding partner of filamin C (FLNc) (Reimann et al. 2020). FLNc belongs to the filamin family of actin crosslinking proteins (Nakamura et al. 2011). The other two members of the filamin family, FLNa and FLNb, are expressed in almost all tissues (Sheen et al. 2002), while FLNc expression is predominantly detected in cross-striated muscles (Mao and Nakamura 2020). Acting in Z-disc formation to connect sarcomeres, FLNc is crucial for myofibrillar development (Dalkilic et al. 2006) and repair of myofibrillar damage (Leber et al. 2016). Similarly to FLNa, binding of FILIP1 leads to FLNc degradation and consequently modulates filamin function (Reimann et al. 2020). Interestingly, Akt- and PKCα-dependent dual-site phosphorylation in the extended basophilic motif protects FLNc from FILIP1-mediated degradation in contracting muscle cells, leading to strongly upregulated expression levels of FLNc and FILIP1, but not of FLNa, during skeletal muscle cell differentiation (Reimann et al. 2020). These results underline that FILIP1 not only mediates the start of cortical cell migration, but is also involved in the expression of MRFs and the differentiation of myoblasts into myotubes.

Interaction of FILIP1 with FLNc in skeletal and cardiac muscle cells plays an important role in Z-disc formation to connect sarcomeres (Thompson et al. 2000; Mao and Nakamura 2020; Reimann et al. 2020). Loss of FLNc leads to severe impairment in myogenesis and in the maintenance of muscle structural integrity, resulting in skeletal myopathy and cardiomyopathy (Dalkilic et al. 2006). Phenotypically, variants in the corresponding *FLNC* gene cause skeletal muscle disorders such as myofibrillar myopathy, distal myopathy and limb girdle muscular dystrophy, whereas associated cardiac defects mainly include dilated, restrictive and hypertrophic cardiomyopathies, and cardiac arrhythmias (Mao and Nakamura 2020). Although a robust *FILIP1* expression has also been detected in heart muscle (Nagano et al. 2002; Sato and Nagano 2005), cardiac investigations of all our five patients revealed no structural or functional anomalies. As all our patients are still children, it seems also conceivable that cardiac anomalies might manifest later in life.

In three patients presenting with a combination of early-onset restrictive cardiomyopathy and congenital myopathy due to de novo* FLNC* missense variants an additional arthrogryposis phenotype was described (Kiselev et al. 2018). In addition, similarly to FLNc, FILIP1 expression is upregulated during myocyte differentiation and repair of myofibrillar damage, suggesting that both genes are activated during myogenesis (Leber et al. 2016; Militello et al. 2018; Reimann et al. 2020). Dual-site phosphorylation protects FLNc from FILIP1-mediated degradation in differentiating myocytes (Reimann et al. 2020). Knockdown experiments additionally revealed that silencing of *FILIP1* inhibits the formation of multi-nucleated myotubes and decreases the expression levels of MRFs *MyoD*, *Myogenin* and *Myolinc* in differentiating myoblasts (Militello et al. 2018). These in vitro studies suggest that homozygous truncating variants in *FILIP1* leading to a premature stop of protein synthesis and probably to a loss of protein function result in significant impairment in embryonic muscle differentiation giving rise to a congenital disorder with multiple joint contractures in our patient cohort.

In summary, we provide clinical and genetic evidence that bi-allelic deleterious variants in the *FILIP1* gene cause a novel autosomal recessive arthrogryposis phenotype with microcephaly. The contractural features in combination with microcephaly in our patients strongly support that FILIP1 plays a significant role in both, skeletal muscle cell differentiation and brain development, during embryonic development. However, further functional studies are necessary to clarify the detailed pathophysiological mechanisms leading to arthrogryposis; this might pave the way for novel treatment strategies.

## Data Availability

The raw exome sequencing data are not publicly available due to privacy or ethical restrictions. Processed genetic data generated or analyzed within this study are available upon request.
